# Drowning in Aboriginal and Torres Strait Islander children and adolescents in Queensland (Australia)

**DOI:** 10.1186/s12889-015-2137-z

**Published:** 2015-08-19

**Authors:** Belinda A. Wallis, Kerrianne Watt, Richard C. Franklin, Roy M. Kimble

**Affiliations:** Queensland Children’s Medical Research Institute, Brisbane, Australia; University of Queensland, Brisbane, Australia; Royal Children’s Hospital, Brisbane, Australia; College of Public Health, Medical and Veterinary Sciences, James Cook University, Townsville, Australia; Royal Life Saving Society Australia, Sydney, Australia

## Abstract

**Background:**

Aboriginal and Torres Strait Islander (Indigenous) children are at greater risk of drowning than other children, however little is known about drowning of Indigenous children. This study identifies the previously unpublished incidence and characteristics of fatal and non-fatal drowning in Indigenous children and adolescents.

**Methods:**

Retrospective data (Jan 2002-Dec 2008) on fatal and non-fatal drowning events among Indigenous and Non-Indigenous Queensland residents aged 0-19 years were obtained from multiple sources across the continuum of care (pre-hospital; emergency department; admitted patients; fatality) and manually linked. Crude incidence rates for fatal and non-fatal events were calculated using population data from the Australian Bureau of Statistics.

**Results:**

There were 87 (6.7 % of all events) fatal and non-fatal (combined) Indigenous drowning events yielding a crude Incidence Rate of 16.8/100,000/annum. This is 44 % higher than the incidence rate for Non-Indigenous children. For every fatality, nine others were rescued and sought medical treatment (average 12 per year). There were no significant changes in Indigenous drowning incidents over the study period. Drowning rates were higher for Indigenous females than males. Overall incidence was higher among Indigenous children and adolescents than Non-Indigenous children for every calendar year and age-group (0-4 years; 5-9 years; 10-14 years) except those aged 15-19 years where no drowning events were recorded for males.

Location of drowning sites was similar in both populations 0-19 years, however there were slight differences in frequency at each of the locations. The three leading drowning locations for Indigenous 0-19 years olds were pool (48 %), bath (21 %) and natural water (16 %), and for non-Indigenous 0-19 years the leading locations were pool (66 %), natural water (13 %) and bath (12 %) (*p* < .01). Except for pool drowning, Indigenous drowning occurred more often in geographic areas of relative disadvantage. Among Indigenous children drowning location varied with age (*p* < .001). Most frequent locations by age were: <1 year bath (71 %); 1-4 years pools (80 %); 5-9 years pools (75 %) and 10-19 years beach/ocean (36 %). Severity of event differed statistically with Indigenous status and by remoteness with all fatal drowning events occurring in Regional or Remote areas, and none in Major Cities.

**Conclusions:**

For every fatal drowning among Indigenous children in Queensland aged 0-19 years there are nine non-fatal events. This previously unreported survival ratio of 9:1 indicates the non-fatal injury burden in Indigenous children aged 0-19 years. Although higher Indigenous drowning rates prevailed, no significant changes over time are concerning. Equally the apparent over-representation of Indigenous adolescent females should be weighed against the absence of drowning among Indigenous male adolescents in the same age group in consecutive years of the study. Further investigation around behaviour and culture may highlight protective factors. Culturally specific prevention strategies which take into account social and demographic indicators identified in this study should be delivered to carers and peers of vulnerable age groups who frequent specific locations. Females, swimming ability, supervision and the young are areas which need to be incorporated into Indigenous-specific interventions for drowning prevention.

## Background

Drowning is among the 10 leading causes of death of children and young people in every region of the world [1]. Drowning varies by age group, but for children and adolescents aged 1-24 years it ranks within the first five causes of death for the Western Pacific Region (which includes Australia) [1]. Indigenous Australians have been found to have a drowning risk 3.6 times that of Non-Indigenous Australians [2]. In Australia drowning is the third leading cause of unintentional injury death [2] and drowning hospitalisation rates are higher for Indigenous people of all ages compared with Non-Indigenous [3].

While it is recognised that Indigenous children suffer a significantly higher burden of morbidity and mortality worldwide [4], in Australia one quarter of all deaths among Indigenous children are due to injury - this is three times the rate for Non-Indigenous children aged 0-17 years [5]. For the same age group in 2003–2007 the mortality rate due to drowning was nearly double that of Non-Indigenous adolescents (2.8/100,000 vs 1.7/100,000 respectively) [5].

National Indigenous drowning data has not been published consistently in the past due to poor documentation of Indigenous status and privacy problems encountered when reporting on sub-groups of populations [4, 6]. Small numbers cause fluctuations in trends and results often require cautious interpretation. For example drowning survivors were thought to be underestimated because of poorly documented Indigenous status where hospitalisation and death rates were reported at the same rate (4.3/100,000) [2, 6]. In particular, variations in ages and reporting deaths or hospitalisations by region make comparisons difficult.

Notwithstanding the state of Queensland having the second largest Indigenous population (146,429 is 6 % of the state) in Australia with almost half aged less than 20 years [7], there has been no previous study on Indigenous drowning in young people in Queensland. This is the first population-based study on drowning where data from multiple datasets have been linked to acquire the most comprehensive identification of Indigenous status, and to capture as many cases as possible of fatal as well as non-fatal drowning among this age group. This study addresses the absence of Indigenous data, identifies the incidence and socio-demographics of drowning mortality and morbidity and describes the characteristics for all age groups including aquatic locations. Comparisons are drawn with the Non-Indigenous population in some instances to better inform prevention strategies.

## Methods

Patient data from multiple portals across the continuum of care were manually linked to collate detailed data on each unique fatal and non-fatal drowning episode in children and adolescents aged 0-19 years in Queensland from 1 January 2002–31 December 2008. Cases were included and categorised as pre-hospital, emergency attendances or admissions if they were present in any of these databases, but were only included once based on their highest level of care. For example, a person recorded in pre-hospital, emergency and admission databases would be categorised as admitted, whereas a person who appeared in pre-hospital and emergency databases would be categorised as emergency. Transfers between hospital facilities were excluded. The final dataset comprised unique drowning events during the study period. For simplicity of reading we refer to Aboriginal and Torres Strait Islander people as Indigenous, and Non-Indigenous for those who did not identify as Indigenous. All people 0-19 years are referred to as “children” and “adolescents” where appropriate. The definition of drowning used in this study is an internationally agreed designation where drowning is acknowledged as a process of respiratory impairment from immersion or submersion in liquid, which can result in death or survival [8]. These analyses used fatal and non-fatal drowning events combined, to avoid the numbers of fatal drowning alone in the Indigenous population being potentially identifying (a requirement of ethics) [9, 10]. “All drowning events” and “total drowning” are terms used to describe fatal and non-fatal drowning events combined.

Data were sourced from hospital based reporting systems Queensland Health Admitted Patients Data Collection (QHAPDC), Emergency Department Information System (EDIS); Surgical and Retrieval Team (SATR); Queensland Injury Surveillance Unit (QISU), Mater Health Services (paediatric and adult), and Queensland Ambulance Service (QAS); Fatal data were provided by National Coronial Information System (NCIS); the Commission for Children and Young People and Child Guardian Child Death Review Unit (CCYPCG); and the Royal Life Saving Society Australia (RLSSA). Details on data linkage and data extraction have been reported elsewhere [11]. Briefly, data on age, gender, severity, (deaths, hospital admission and pre-hospital/emergency presentation), drowning location of event, geographical event location, event time, and day of week, Socioeconomic Index for Areas (SEIFA) [12] (based on residential postcode) and Accessibility Remoteness Index of Australia (ARIA) [13] (based on event postcode) were collected. Data are categorised under the headings of Major Cities, Inner and Outer Regional, and Remote and Very Remote based on the accessibility to services from these locations.

Self-identified Indigenous status is recorded routinely in all datasets accessed for this study except QAS. Country of birth and Medicare eligibility are routinely collected in emergency and hospital admission data. These variables were cross matched to validate Indigenous status by excluding people born overseas or for those who were non-resident and therefore not eligible for Medicare. For the purposes of this study Indigenous status was allocated using adapted algorithms [14] where Indigenous status was allocated based on the number of times the status values were recorded in individual datasets. If non-Indigenous is greater than Indigenous, status = Non-Indigenous; if Non-Indigenous is less than Indigenous, status = Indigenous; if Non-Indigenous = Indigenous, status = Non-Indigenous; if Non-Indigenous is missing or unknown and Indigenous is missing or unknown, status = unknown. Where two records indicated Non-Indigenous and Indigenous = Indigenous (only two cases). The term “Non-Indigenous” applies to those who identified as not being Aboriginal and/or Torres Strait Islander, and following published guidelines [15], 246 patients were excluded from analyses where Indigenous status was not recorded in any dataset. Site of immersion data were ascertained from police and coroners’ reports (for deaths), and for non-fatal drowning, mined from variables such as triage text, presenting problem, place/location definitions, activity description, major injury factor descriptions, and the pick-up location where an ambulance attended.

To calculate crude Incidence Rates (IRs) for Indigenous and Non-Indigenous children in each age group, the estimated Indigenous and Non-Indigenous population at 2006 [16] for each age group was used as the population denominator for the specified age group for every year from 2002–2008. This is because while population data for each year of age are available for each calendar year for non-Indigenous Queensland residents [17], these data are not available for Indigenous children. Queensland residents were identified by postcode of usual residence and crude IRs were calculated for fatal and non-fatal drowning events (total number of events divided by population). The ABS provides estimated summary population data for Indigenous and Non-Indigenous children, by age-group (0-4 years; 5-9 years; 10-14 years; 15-19 years), for the year 2006 [16].

Crude IRs are presented per 100,000 population together with 95 % Confidence Intervals (CI), stratified by age group and gender for every calendar year from 2002–2008, for total drowning events, fatal events and non-fatal events. Chi square test for trends over time were computed using Centers for Disease Control and Prevention Epi Info™ 7.1.2.0. Relative Risks (RR) and 95 % Confidence Intervals were calculated using IBM SPSS Statistics for Windows, Version 22.0. Armonk, NY: IBM Corp released 2013. Descriptive analyses (primarily chi-square tests) were also conducted to determine whether there were cultural variations in characteristics of drowning events. Fisher’s Exact test was used where assumptions of chi-square tests were violated.

Ethics and approvals were sought and granted from Children’s Health Services District (Royal Children’s Hospital Human Research Ethics Committee HREC/09/QRCH/38; Royal Children’s Hospital Institutional Approval; University of Queensland Medical Research Ethics Committee #2009001463; Mater Health Services Human Research Ethics Committee #1446E; and National Coronial Information System #CF/07/13729 (2007–2010), #CF/10/25057 (2010–2013), #CF/13/19798 (2013–2016). Director General approval was granted through Public Health Application, Queensland Health 16/3/2010 Ref RD002254.

Custodian approvals were granted from Royal Life Saving Society Australia, Commission for Children and Young People and Child Guardian, Queensland Ambulance Service.

## Results

### Descriptive characteristics

A total of 1299 cases of fatal and non-fatal drowning aged between 0-19 years were identified in the seven years 2002–2008 in Queensland. Indigenous status was recorded for 81 % (*N* = 1053) of cases. Results are presented only for cases for which data on Indigenous status was recorded. Nineteen per cent (*n* = 246) of cases were excluded from analyses on this basis. Where Indigenous status was known, there were 87 Indigenous and 966 Non-Indigenous drowning events over the seven years of the study, yielding an annual average of 12 and 138 drowning incidents respectively. Indigenous children represented 6.7 % of the total drowning events. The survival to death ratio for Indigenous drowning in children is 9:1.

#### Severity and cardiopulmonary resuscitation (CPR)

Comparative data for severity and CPR are described in Table 1. Overall, Indigenous children constituted 7 % of presentations not admitted, 9 % of admitted patients and 8 % of fatalities. Approximately 30 % of cases had first responder attempts at CPR. Proportionately, CPR was attempted in both populations at the same frequency (Indigenous 29 % and Non-Indigenous populations 30 %).

**Table 1:** Drowning 0-19 years by severity, gender and Australian Indigenous status, Queensland 2002-2008

	0-19 years Non-Indigenous (*n* = 966)	0-19 years Indigenous (*n* = 87)	*P* value
	%	%	
Fatal	11 % (110)	10 % (9)	*X* ^2^ = 0.42 df2 p = .810
Non-Fatal	90 % (856)	90 % (78)	
*Admitted*	68 % (656)	71 % (62)	
*Not Admitted*	21 % (200)	19 % (16)	
Male:Female	1.6:1	1:1	*X* ^2^ = 4.78 df1 p = .029
Received CPR	30 % (29)	29 % (25)	

#### Location

Location could not be identified for 116 (11 %) drowning events where Indigenous status was known (Table 2). Even though the top three ranked locations were similar sites for both Indigenous and Non-Indigenous populations, the proportion by drowning location varied significantly with Indigenous status (*X*^2^ = 11.31 df4 p = .026), with most variation observed in the 0-4 years old group. The adolescent age groups 10-14 years and 15-19 years each had drowning events at only two locations.

**Table 2:** Drowning location by Australian Indigenous status and age group 0-19 years, Queensland 2002–2008 (*N* =1053)

	Non-indigenous	Indigenous	
	Rank of locations by % of drowning events (n)	Rank of 5 locations by % of drowning events (n)	*P* value^a^
	(*n* = 859)	(*n* = 78)	
0-19 years	1. Pools 64 % (549)	1. Pools 47 % (37)	*X* ^2^ = 10.82 df3 *p* = .013
	2. Natural water 15 % (133)	2. Bath 21 % (16)	
	3. Bath 12 % (103)	3. Natural water 17 % (13)	
	4. Coast 9 % (74)	4. Coast 15 % (12)	
AGE GROUP	Leading locations by % of drowning events within age group (n)	Leading locations by % of drowning events within age group (n)	
	(*n* = 859)	(*n* = 78)	
	*X* ^2^ = 228.2 df = 6 < 0.001^b^	*X* ^2^ = 15.27 df = 6 *p* < 0.01^b^	
0 to 4 years (*n* = 669)	1. Pool 70 % (426)	1. Pool 52 % (30)	
	2. Bath 16 % (97)	2. Bath 26 % (15)	
	3. Natural water 12 % (74)	3. Natural water 12 % (7)	
	4. Beach or ocean 2 % (14)	4. Beach or ocean 10 % (6)	
<1 year	1. Bath 67 % (55)	1. Bath (np)	
	2. Pool 21 % (17)	2. Pool (np)	
	3. Natural water (np)	3. Natural water (np)	
	4. Beach or ocean (np)		
1 to 4 years	1. Pool 77 % (409)	1. Pool 60 % (29)	
	2. Natural water 12 % (65)	2. Bath 15 % (7)	
	3. Bath 8 % (42)	3. Natural water / Beach or ocean 25 % (12)	
	4. Beach or ocean 3 % (13)		
5 to 9 years (*n* = 118)	1. Pool 75 % (80)	1. Pool (np)	
	2. Natural water 15 % (16)	2. Beach or ocean (np)	
	3. Beach or ocean (np)	3. Natural water (np)	
	4. Bath (np)	4. Bath (np)	
10 to 19 years (*n* = 150)	1. Beach or ocean 36 % (50)	1. Beach or ocean (np)	
	2. Pool 31 % (43)	2. Natural water (np)	
	3. Natural water 30 % (43)	3. Pool (np)	
	4. Bath 3 % (5)		

^a^frequency of specific age group at four locations (Beach or ocean, Pools, Natural water (inc Other), and Baths) comparing Indigenous status. Note: Location was unknown in 116 (11 %) of cases where indigenous status was known; ^b^this chi-square relates to comparison between age groups 0–4, 5–9 and 10-19 years and location for Indigenous and Non-Indigenous separately; np = not publishable due to small numbers <5 and/or being potentially identifiable

Percents and numbers have not been presented intentionally, where small numbers may breach our ethics and access compliance, consequently percents may not add to 100 % within age groups

Gender differences were observed for drowning location in Indigenous children, but these were not significant (*X*^2^ = 7.55 df3 *p* = .056). Compared to all other locations, for males the most frequent place was pools (62 %) (*X*^2^ = 5.51 df1 *p* = .019), whereas for females, the most frequent location was baths (75 %) (*X*^2^ = 4.30 df1 *p* = .038). Pool related drowning events were more common at private residences (54 % Indigenous and 68 % Non-Indigenous) compared to public pools (27 % and 17 % for both Indigenous and Non-Indigenous children respectively). While the proportions were different this was not significant, (*X*^2^ = 2.86 df1 *p* = .091).

#### Indigenous and Non-indigenous drowning by age group

The proportions of each age group involved in drowning were similar between the two populations (*X*^2^ = 4.69 df3 *p* = .196). The majority of drowning events occurred in toddlers aged 0-4 years who made up 76 % and 72 % of Indigenous and Non-Indigenous drowning incidents respectively. Proportionally fewer Indigenous 15-19 year olds were involved in drowning incidents than their Non-Indigenous counterparts (3 % vs 11 %). Drowning locations were similar between the two populations although Indigenous children 0-4 years (52 %) had fewer events in pools than Non-Indigenous children (70 %) of the same age, and more drowning episodes in baths (26 % vs 17 %) (*X*^2^ = 18.02 df4 *p* = .001).

#### Indigenous drowning location by age group

There were significant differences for location by age group among Indigenous children. (*X*^2^ = 15.27 df6 *p* < .01). Those aged 0-4 years were more likely to have suffered a bathtub drowning than their older counterparts 5-19 years (25 % vs 5 %). All but one bathtub drowning occurred in 0-4 years and most (50 %) were aged <1 year. Just over half (52 %) of drowning events for younger children 0-4 years occurred in pools compared with 35 % for 5-19 years. Natural water and the beach or ocean (30 % each) were next the most likely locations for children 5-19 years.

#### Remoteness

**Fig. 1:**
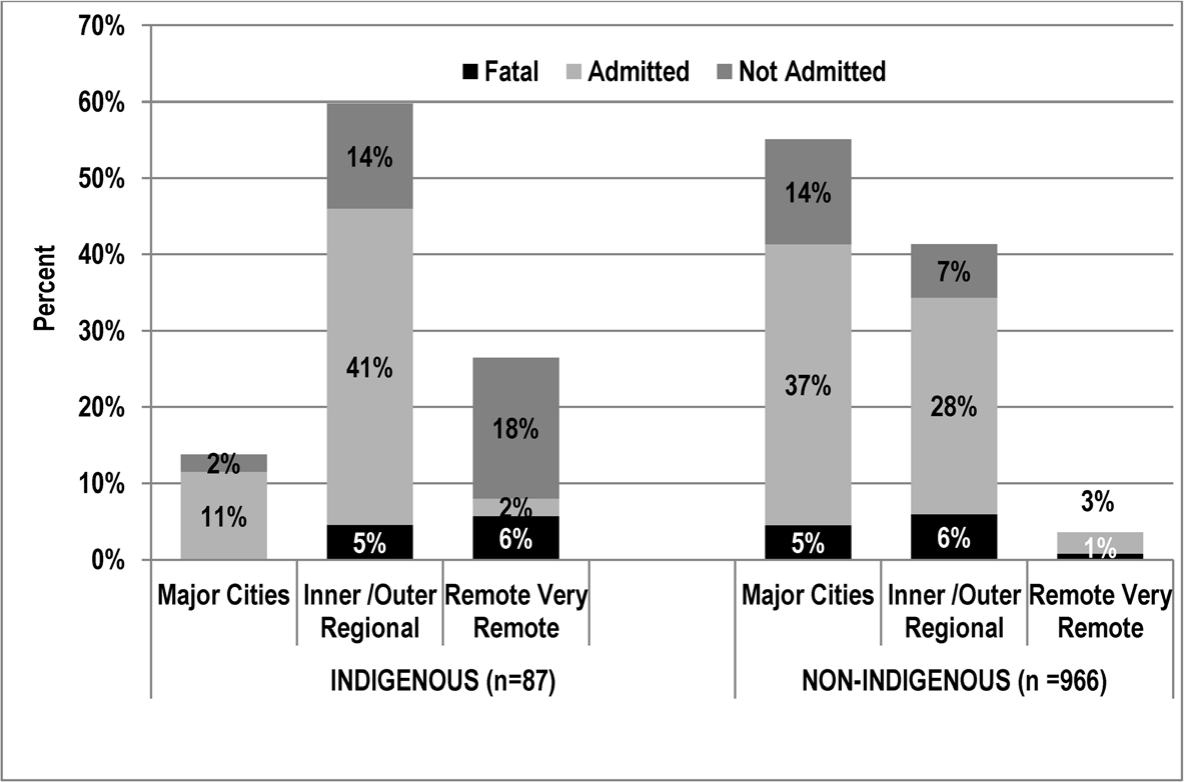
Geographical location of drowning events by Indigenous status and severity 0-19 years, Queensland, 2002–2008 (*n* =1053)

The geographical location of drowning event (defined by ARIA) is shown in Fig. 1 (stratified by Indigenous status and severity). (Remoteness categories for Inner and Outer Regional were grouped together, as were the categories Remote and Very Remote). Drowning differed significantly by remoteness (ARIA) of event and Indigenous status (*X*^2^ = 85.09 df2 *p* < .001), with proportionally more Non-Indigenous events occurring in Major Cities (98 %) and Regional areas (89 %). Drowning severity also differed by remoteness with all fatal drowning events occurring in Regional or in Remote areas for Indigenous children, compared with the majority of Non-Indigenous drowning occurring in Major Cities. For the Indigenous population over half of fatalities (56 %) occurred in remote areas (notably, there were none in Major Cities) whereas the majority of non-fatal drowning occurred in Regional areas. The inverse was seen in non-Indigenous children. The majority of drowning events for the Indigenous population occurred in pools in Major Cities (75 %) and Regional (52 %) areas, but in Remote (30 %) areas the beach or ocean was the most frequent location. For Non-Indigenous children, pools were the most frequent drowning location for all geographic regions.

#### Season, weekday and time

The warmer months from October through March showed consistently higher frequency patterns for all drowning deaths (78 %), (*X*^2^ = 371.84 df = 1 *p* < .001) this is not significantly different between Indigenous (80 %) and Non-Indigenous (77 %) populations (*X*^2^ = .45 df1 *p* = .502). Sunday, Monday and Friday were the days that drowning events most commonly occurred for Indigenous children, but this was not significantly different to Non-Indigenous children (*X*^2^ = 8.86 df6 *p* = .182) (Table 3). The most critical times for drowning among the Indigenous population were between 12:00–15:00 (28 %) and 15:00–18:00 (38 %). The location of drowning event varied by time of day for Indigenous children (*X*^2^ = 13.68 df9 p = .050). The most frequent time of day for all locations was between 12:00–18:00 for both populations, but 20 % of Indigenous bath drowning events occurred between 06:00–12:00 (vs 6 % of pool drowning) and 17 % of pool drowning occurred between 18:00–21:00 (vs 7 % of bath drowning).

**Table 3:** Season, day of week and time of incident by Indigenous status, Queensland, 2002–2008 (*N* = 1053)

	Day of incident		
Season	Indigenous	Non-Indigenous	Total
Cool/Dry Apr - Sept	20 % (17)	23 % (219)	22 %
Warm/Wet Oct-March	80 % (70)	77 % (747)	78 %
Day of Week			
Sunday	22.8 % (19)	20.7 % (200)	20.8 %
Monday	18.4 % (16)	11.2 % (108)	11.8 %
Tuesday	9.2 % (8)	12.1 % (117)	11.9 %
Wednesday	12.6 % (11)	10.6 % (102)	10.7 %
Thursday	6.9 % (6)	11.0 % (106)	10.6 %
Friday	16.1 % (14)	12.0 % (116)	12.3 %
Saturday	14.9 % (13)	22.5 % (217)	21.8 %
Time of Day			
06:00–11.59	9.2 % (8)	16.9 % (164)	16.4 %
12:00–14:59	27.9 % (24)	25.2 % (243)	25.4 %
15:00–17:59	37.9 % (33)	25.2 % (340)	35.4 %
18:00–20:59	13.8 % (12)	15.0 % (145)	14.9 %
21:00–00:00	6.9 % (67)	6.8 % (6)	7.0 %

There were 11 events where time was unknown

#### IRSAD

Against the Index of Relative Socioeconomic Advantage and Disadvantage (IRSAD) [12] the majority (58 %) of Indigenous children involved in drowning events resided in areas categorised as being more disadvantaged (IRSAD 1-5 [50]). This is true for only 28 % of Non-Indigenous children (*X*^2^ = 32.09 df1 *p* < .001).

More than three quarters (77 %) of all drowning incidents that occurred in pools were at residents where households were in the highest 50 % of IRSAD (decile 6–10) indicating a relative lack of disadvantage and greater advantage in general. For Indigenous children drowning location differed by IRSAD (*X*^2^ = 13.77 df3 *p* = .003). For Indigenous children, 62 % of drowning events that occurred in pools involved children categorised as advantaged (IRSAD 6–10), whereas for all other drowning locations (beach: 92 %, natural water 77 %, baths 63 %), the majority of children were categorised as disadvantaged (IRSAD 1–5): beach 92 %; natural water 77 %; and baths 63 %.

### Crude incidence rates

**Fig. 2:**
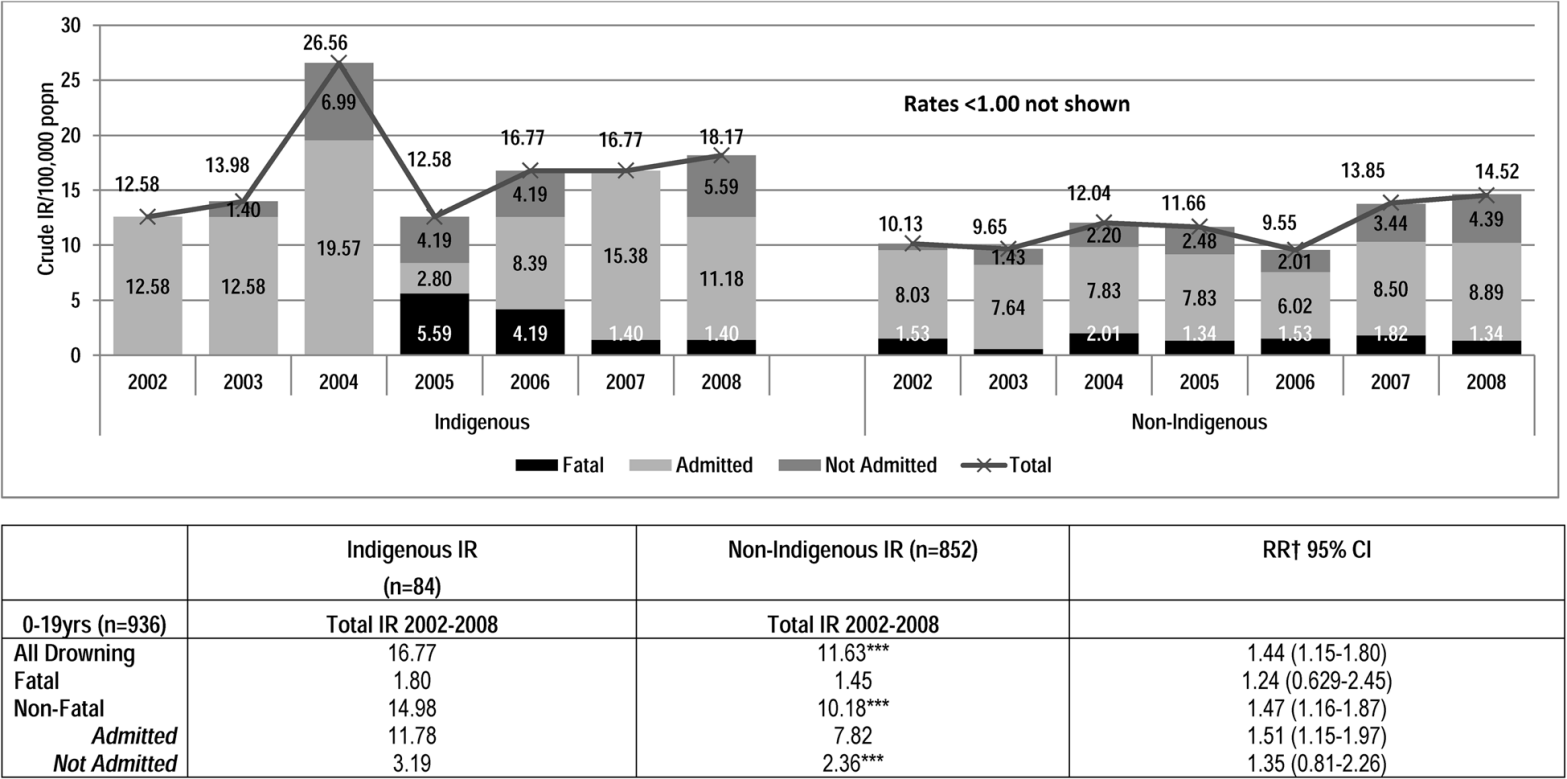
Crude incidence rates for Indigenous and Non-Indigenous 0-19 years by severity and gender, Queensland 2002–2008

Crude IRs stratified by severity of drowning events (Fig. 2) for 0–19 year olds show Indigenous drowning rates were higher for all types of drowning episodes for every year of the study. The highest number (19) of Indigenous drowning events was in 2004 and varied between 9 and 13 incidents for the other years. The highest number of Non-Indigenous drowning events was 152 in 2008. The non-fatal drowning rates were 47 % higher among Indigenous children than Non-Indigenous children and a risk of any drowning events was 1.44 times that of their Non-Indigenous counterparts (RR = 1.44 95 % CI = 1.15-1.81).

Although Indigenous drowning rates were higher overall than Non-Indigenous rates, drowning incidence increased over the study period for both groups at a similar magnitude 44 % (12.58 in 2002 to 18.17 in 2008) in Indigenous children (not significant) and 43 % (from 10.13 to 14.52) among Non-Indigenous children (this was significant *X*^2^ = 11.68; *p* < .001). There were no significant changes over time in any Indigenous drowning rates, most probably due to small numbers and the unusual peak that occurred in 2004. Significant increases were observed in non-fatal and non-admitted rates for Non-Indigenous males, rates.

**Fig. 3:**
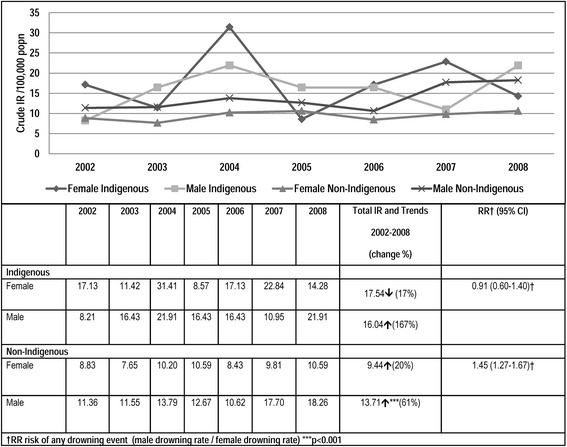
Crude incidence rates all drowning among Queensland residents by calendar year, Indigenous status and gender 2002–2008

Highest rates were observed in Indigenous females rather than males, (Fig. 3) however, these rates did not increase over time as did Indigenous male rates, (though not significantly) and rates for both male and female Non-Indigenous children. Rates were higher in Indigenous females than males for four of the seven years. Among Indigenous males drowning incidences increased by a factor of 2.7 over the period. Total drowning rates were higher in female Indigenous children than male Indigenous children, but the opposite was true for Non-Indigenous children. Indigenous females had a risk 1.86 times that of Non-Indigenous females (RR = 1.86 95 % CI 1.35-2.55) whereas Indigenous males was 1.17 times Non-Indigenous males (RR = 1.17 95 % CI = 0.85-1.61).

### Incidence by age group and gender

Data on trends over time by age group and gender are not shown due to small numbers, however summary data are described below. Rates were higher for Indigenous children in all age groups except 15-19 year old males (Table 4), where there was no drowning event recorded during the study period, resulting in a 31 % less risk of drowning than their Non-Indigenous counterparts (RR = 0.69 95 % CI = .218-2.19). Indigenous females aged 0-4 years incurred the highest rates in any year. For Indigenous females 5-9 years and Indigenous boys aged 10-14 years, there were no recorded drowning events in five of the seven years of the study period. For 10-14 years old Indigenous females there were no recorded fatal or non-fatal drowning events in three of the seven years of the study period.

**Table 4:** Crude incidence rates for total drowning by age group, and gender, Queensland 2002–2008

	Indigenous			Non-Indigenous			RR^a^ (95 % CI)		
All drowning events	Male	Female	Total	Male	Female	Total	Males	Females	Total
0-4 years	47.73	50.14	48.92	42.97*	29.60	36.48	1.11 (0.77-1.59)	1.70 (1.18-2.43)	1.34 (1.04-1.73)
5-9 years	8.99	4.63	6.84	5.85**	3.42**	4.66***	1.54 (0.66-3.56)	1.35 (0.41-4.44)	1.47 (0.74-2.92)
10-14 years	4.45	6.35	5.37	2.77	2.92	2.84	1.59 (0.48-5.23)	2.18 (0.76-6.22)	1.89 (0.86-4.16)
15-19 years	0.00	5.82	2.83	5.00	3.16	4.10	-^b^	1.84 (0.56-6.05)	0.69 (0.22-2.19)
0-19 years	16.04	17.54	16.77	13.71***	9.44	11.63***	1.17 (0.85-1.61)	1.86 (1.35-2.55)	1.44 (1.15-1.80)

^a^Relative Risk (RR) risk of total Indigenous drowning relative to total Non-Indigenous drowning by age group

*p < 0.05; **p < 0.01; ***p < 0.001 used to denote significance for change over the seven year period

^b^RR (and 95 % CI) has not been calculated because one of the incidence rates is equal to 0

Increases over time were observed in the Indigenous population for total drowning in 0-4 years (50 % increase). Increases were also seen in Indigenous males 0-4 years (150 %) and 10-14 years, as well as Indigenous females 5-19 years. However, no significant changes over time occurred in rates in any Indigenous age group or gender over the study period.

## Discussion

The effectiveness of data linkage across the continuum of care has demonstrated that a most accurate assessment of drowning incidence is possible. These data reveal a drowning rate of 16.8/100,000 per annum for Indigenous children 0-19 years, which is 1.44 times higher than the incidence rate for Non-Indigenous children. Overall incidence was higher among Indigenous children and adolescents than Non-Indigenous children for every calendar year and age group (0-4 years; 5-9 years; 10-14 years) except those aged 15-19 years. Indigenous children have been identified as at risk [18], and in this study a national drowning mortality rate of 1.8 compares favourably against a national figure of 2.8 among Indigenous children 0-17 years in 2003-2007 [5]. Furthermore the fatality risk ratio for Queensland is an improvement on the national figure (1.2 vs 1.7 respectively) [5]. However, this study shows that considering death data alone is not necessarily a clear view of the drowning burden on the Indigenous community or the health system. There is room for improvement, particularly where hospitalisation rates for injury (which includes drowning) in Indigenous children showed no significant change from 2004–05 to 2009-10 [19].

A drowning survival ratio for Queensland Indigenous children was 9:1. That is, for every fatality nine others are rescued and survive. On average one Queensland Indigenous child or adolescent was involved in a drowning episode each month. Indigenous children 0-19 years represent 6 % of the Queensland population, and this study found they represent 6.7 % of drowning events. However, if 246 events with unidentified status were not included this figure would indicate over-representation at 8.3 % [15].

Indigenous children have a significantly higher risk (44 %) of drowning than Non-Indigenous children. Drowning rates increased over the seven year period by 43 % in Indigenous children. No significant changes in rates were observed during the study period for Indigenous children overall, or for gender and age subgroups. This is of concern because it demonstrates that there was no reduction in drowning in the Indigenous population aged 0-19 years over the study period, and highlights the need for targeted culturally appropriate drowning prevention programs.

Drowning has the heaviest toll on the young [1, 20–22], and Indigenous toddlers have been previously identified as the most at-risk group [21–23]. The rates identified in this study for fatal and non-fatal drowning at 48.92/100,000 and 36.48/100,000 for Indigenous and Non-Indigenous toddlers respectively concur with those findings. Indigenous toddlers had a risk 1.34 times that of Non-Indigenous, although Indigenous toddlers had a (non-significant) increased drowning over time (*p* > 0.05). Compared to these findings however, national data for non-fatal hospitalisations for Indigenous children 0-4 years were more than double that of their Non-Indigenous counterparts (34/100,000 vs 14/100,000) [3]. These results are also in contrast to three previous studies from the Northern Territory where fatal Non-Indigenous toddler drowning was at higher rates than Indigenous [24–26]. Comparisons are problematic however, with rates calculated for fatal, admissions or fatal and non-fatal combined. An evaluation of any drowning prevention interventions which may be introduced as a result of these data would support and further inform drowning prevention in this age group.

There is no apparent rationale other than random variability for the 2004 peak in Indigenous drowning, where Indigenous female non-fatal drowning effectively doubled the average numbers and was contributed to by higher than average Indigenous male drowning and toddlers. Prevention interventions and weather were ruled out. It also should be noted that there were no fatalities among the 19 female events. Whereas being male is a risk factor in most injury or drowning scenarios [1], Indigenous females 0-19 years drowned at higher rates than their male counterparts. This finding has not been found elsewhere; however this apparent incongruity was more likely due to no drowning being recorded for males 15-19 years over the seven years of the study. Female drowning differed by age group, the 15-19 years age group incurred higher rates than their male counterparts, and this gender anomaly was consistent with national data for females 5-14 years where rates were higher as well [3]. Drowning rates in Indigenous adolescents were half (45 %) that of Non-Indigenous 15-19 years, and this is certainly a factor worthy of further investigation both in terms of culture and behaviour to identify protective factors. Injury prevention approaches should focus on the activities of females for swimming ability and supervision around pools and bath time. Similarly, investigation of potential protective factors for adolescent males would inform prevention strategies.

Very little prevention work has been undertaken around adolescent drowning previously. West Australian Indigenous children 0-14 years fatally drowned at twice the rate of Non-Indigenous in the early 1990’s [22], and in the Northern Territory Indigenous fatal rates varied from 9 % higher (5.4/100,000 1983–1998) to Non-Indigenous rates being 34 % higher (9.2/100,000 in 1998) [24, 25]. Adolescent behaviour may play a role in fewer drowning incidents in this age bracket. Indigenous numbers in the adolescent age groups are relatively low and may be linked to cultural practices. Roaming further from home and with less supervision are life skills which require to be learned earlier in some cultures than others, and for those who live in less urbanised areas, this freedom comes more easily. Similarly, protective factors may be associated with groups of adolescents looking out for each other.

The older adolescent age group will require culturally appropriate interventions to be devised which are specific to regions as well as aquatic locations. Incorporating links to land and community will assist success such interventions [27]. The method of delivery is particularly important for these populations and will require appropriate consultative input [28, 29].

The top three ranked locations although the same in each of the populations were statistically different with Indigenous drowning less frequent in pools and more frequent in baths. These findings are consistent with earlier research from the 1970’s [30–32] and also the unique drowning pattern found in the Northern Territory where lower pool drowning rates were found in Indigenous children 0-4 years than in Non-Indigenous children the same age [25, 26]. The authors attributed this over-representation of Non-Indigenous children to lifestyle affluence and access to pools. Interestingly, findings in this study agree with pool drowning being connected to more advantaged residences [12], but that Indigenous children also have access (54 %) to such pools as well, and consequently share the drowning risk. Better supervisor and lifeguard vigilance at public pools could potentially reduce the 27 % of drowning events at those locations for Indigenous children. All other Indigenous drowning locations were in areas at the most disadvantaged end of the scale pointing to a need for prevention education for this demographic.

Our geographic data indicate that Regional areas and Remote or Very Remote locations have proportionately more (84 %) Indigenous drowning events than do Major Cities. Education interventions must reflect appropriate and current demographics and target a broad cross-section of the population to be effective. It may be that new media should be explored as a method of water safety programs to small populations across vast distances.

There were differences in drowning locations by age group. Parents and carers of infants less than one year old should be reminded never to leave baby alone in water or in the care of another sibling. Of concern is the high number of drowning incidents in pools in both Indigenous and Non-Indigenous populations, especially those aged less than five years. National data on injury hospitalisation are similar indicating the majority of 0-4’s non-fatal drowning is related to pools or bathtubs [3]. Queensland has the highest pool ownership in Australia at one pool per six households [33, 34]. Data on presence or adequacy of pool fencing was not available for this population, however pool fencing has been proven successful in preventing unintended access to pools of young children and coupled with adequate enforcement, such as Queensland has implemented in 2010 is necessary to make this strategy effective [35–38]. Other life saving measures such as first responder attempts at CPR were collected from police and paramedic reports and while proportions are equal amongst the two populations, a 30 % uptake indicates room for improvement in learning or attempting CPR. This may be of greater importance to the Indigenous population where the majority of incidents occurred in Regional and Remote areas and where help may take longer to arrive.

### Strengths and limitations

The detail in the data from this study provides the first evidence of fatal and non-fatal drowning incidence among Indigenous and Non-Indigenous children in Queensland.

This is the first study in which state-wide data across the continuum of care (pre-hospital to fatality) have been linked to estimate the magnitude and nature of drowning events among Indigenous Australian children, over a significant period of time, and the authors believe it is the most comprehensive and accurate data on Indigenous drowning in Australia to date. The inclusion of data from sources such as pre-hospital (QAS) and QISU (dedicated injury surveillance data) represent a significant improvement over previous studies (especially in relation to details of non-fatal drowning events (for example, drowning location and time of day).

It is acknowledged that Indigenous status has not been well documented in health records [4, 6], however, accuracy of Indigenous identification from public hospital records in Queensland was 87 % in 2011–12. For Australia-wide data in 2007–2008 this estimate was 89 % [39, 40]. The authors therefore acknowledge that data on Indigenous status may be under-reported by 11-13 %, and that this along with 19 % of cases which were excluded due to unknown status may create a bias in the data. However, there is no way of knowing what proportion of children who were excluded from these analyses due to unknown Indigenous status were Indigenous. It is also possible that some of the drowning events involving Indigenous children during the study period were misclassified into the non-Indigenous group, due to reluctance to report true Indigenous status by carers, or due to errors by medical staff. In addition, identification of Indigenous status is not routinely or consistently collected in all data sets. This information is not collected in QAS data so drowning events which had prehospital attendance only were not included in this study. For all these reasons, it is likely that these results are an underestimate of Indigenous involvement in drowning events.

It appears that the absence of drowning events in the Indigenous 15-19 years males is accurate, as there is no apparent anomaly in the data collection for the same age-group in the Non-Indigenous population. The authors can only assume that if there were drowning events that they were not serious enough to have sought medical attention.

## Conclusions

Fatal and non-fatal drowning incidence among Indigenous and Non-Indigenous children in Queensland is presented for the first time, where the Indigenous population rate was 1.44 times higher. Among the Indigenous population, for every fatality, nine children survived. The over-representation of young Indigenous children and females over the study period will need to be addressed in a culturally appropriate manner at many levels to reduce drowning rates. Equally the apparent over-representation of Indigenous adolescent females should be weighed against the absence of drowning among Indigenous male adolescents in the same age groups in consecutive years of the study. Investigation around behaviour and culture may highlight gender-related protective factors. The quality of data from this study identifies risk factors and gives good insight for interventions to target specific socio-demographics, age and gender groups. Regional areas and Remote and Very Remote communities within Queensland and pool and bathtub safety for the young are particular prerequisites.

## Abbreviations

A&TSI: Aboriginal and Torres Strait Islander
ABS: Australian Bureau of Statistics
ARIA: Accessibility Remoteness Index of Australia
CPR: Cardiopulmonary Resuscitation
IR: Incidence Rates
IRSAD: Index of Relative Socioeconomic Advantage and Disadvantage
np: not publishable
NSW: New South Wales
WA: Western Australia
Qld: Queensland
Tas: Tasmania
NT: Northern Territory
SA: South Australia
SEIFA: Socio-economic Index for Areas
